# News and Coming Events

**Published:** 1908-11-07

**Authors:** 


### News and Coming Events.

An official notice in the *London Gazette*, dated October 20, 1908, states this his Majesty the King has been pleased to approve of the disbandment of the Royal Army Medical Corps (Militia).

Mr. P. F. Willert has been unanimously elected Treasurer of the Radcliffe Infirmary and County Hospital, Oxford, in succession to the Provost of Worcester College, who has lately resigned.

Upon the list of Fellows recommended by the President and Council of the Royal Society for election into the Council for the year 1908-9 appear the names of the following members of the medical profession: Professor John Rose Bradford, M.D., D.Sc. (for the post of Joint Secretary), Professor James Cossar Ewart, M.D., Dr. John Scott Haldane, M.D., and Dr. Frederick Walker Mott.

Sir Edward Grey and the German Ambassador signed an agreement on Tuesday, October 27, by which England and Germany agree to co-operate in combating the sleeping sickness in their East African possessions. The co-operation will take the form chiefly of exchanging reports of cases, and in arranging for the destruction of animals whose blood supplies the virus which is transmitted to human beings by flies or mosquitoes. Measures are also to be taken to prevent the passage of infected individuals across the frontier from one Colony to the other.

The annual dinner of the medical staff and students of the Central London Throat and Ear Hospital was held at the Trocadero Restaurant on October 16, Dr. Dundas Grant in the chair. The principal guests were Dr. John Macintyre, of Glasgow, who delivered the inaugural address at the beginning of the winter course of lectures; Dr. Otto Freer, of Chicago, together with various members of the staffs of all the throat and ear hospitals in London.

A number of French medical practitioners, representing the association known as the "E.M.I." (Etudes Médicales Internationales), have lately been paying a short visit to London. On October 29 they visited the Royal College of Surgeons, and were conducted over the several departments by the officials of the College. A pamphlet which had been translated into French, giving a short account of the Museum, was much appreciated by the visitors. Later in the day the party was entertained at luncheon by Sir Dyce Duckworth.

The Chairman and committee of the Seamen's Hospital Society were "At Home" at the *Dreadnought* Hospital, Greenwich, on the afternoon of Wednesday, October 28. Subscribers and others had an opportunity of inspecting the recent alterations and improvements in the west wing of the building, which, thanks to a grant from the King's Hospital Fund, has been almost entirely remodelled. The old three-bedded wards has been done away with, and a smaller number of well-lighted and commodious wards have been provided, while the floors, which had become worn, have been laid with a new plastic composition of a colour which harmonises with the pale green of the walls. It is hoped, as funds permit, to treat the other wing in similar fashion. The guests were received by Mr. Percival A. Nairne, Chairman of the Society, and Captain F. M. Ommanney, R.N., Deputy Chairman, and among those present were Admiral Holland and Mr. Daniel Danielsen, Consul-General for Sweden.

Principal Donald MacAlister, M.D., the President, will preside at the next session of the General Medical Council, which commences on November 24.

The Bisset Hawkins gold medal of the Royal College of Physicians of London has been awarded to Sir Shirley Murphy, medical officer of health of the County of London, for his distinguished services in the cause of public health.

A Royal Medal has been awarded by the President and Council of the Royal Society to Dr. Henry Head, on the ground of his researches on the relations between the visceral and somatic nerves, and on the functions of the afferent nerves.

His Majesty the King has expressed his willingness to open the new Manchester Infirmary building next July, provided arrangements can be made for him to do so without interfering with his presence at the review of the Territorial Army, which is the primary object of his Majesty's visit to Lancashire.

A Court of Governors of St. Bartholomew's Hospital was held on Thursday of this week to elect a Treasurer in the room of Lord Ludlow, recently resigned. It was previously announced that Lord Sandhurst would be nominated for the position, but the result of the election has not been received at the time of going to press.

The Faculty of Medicine of the University of London has elected as its new Dean Professor Sidney Martin, M.D., F.R.S., and the Senate of the University has appointed Mr. E. A. Minchin, M.A., Professor of Protozoology, to represent the University at the Darwin centenary celebration to be held at Cambridge in June 1909.

The names of Dr. J. Carswell and Dr. T. S. Clouston appear upon the Departmental Committee which the Secretary of Scotland has appointed, to inquire into the operation in Scotland of the law relating to inebriates and to their detention in reformatories and retreats, and to report what amendments in the law and its administration are desirable.

The Mayor and Borough Council of Shoreditch on October 29 visited the Queen's Hospital for Children, Hackney Road, and inspected the wards and various departments, which have been greatly improved in recent years. The Mayor has opened a borough fund for contributions towards the sum of £8,000, which is urgently needed to free the hospital from debt.

Dr. Thomas Renton Elliott, M.D. and M.A., of Trinity College, Cambridge, has been elected to a Fellowship at Clare College. Dr. Elliott graduated in 1900, being placed in the First Class of the Natural Science Tripos, Part I., and in the following year he gained a First Class in Part II. of the same Tripos. He is at present on the resident medical staff of University College Hospital.

The Royal Colleges of Surgeons and Physicians have decided to add the Victoria Infirmary, Glasgow, to the list of general hospitals recognised by the Board. The colleges have also decided to add the University of Nebraska, United States, to the list of universities at which the curriculum of professional study required for the diplomas of the two colleges may be pursued, and whose graduates may be admitted to the final examination of the examining Board, on production of the required certificates of study.

